# Survival trends in heart transplant patients supported on ECMO and IABP: A 10-year UNOS database analysis

**DOI:** 10.1016/j.ijcha.2024.101486

**Published:** 2024-08-13

**Authors:** Chidiebere Peter Echieh, Mohammad Hamidi, Michael P. Rogers, Deepak Acharya, Toshinobu Kazui, Robert L. Hooker

**Affiliations:** a Department of Surgery, University of Calabar, Calabar, Nigeria; b Division of Cardiothoracic Surgery, Department of Surgery, University of Arizona, Banner Tucson, Tucson, AZ, United States; c Department of Surgery, University of Arizona, Tucson, AZ, United States; d Department of Surgery, University of South Florida Morsani College of Medicine, Tampa, FL, United States; e Banner University Medical Center Tucson, Tucson, AZ, United States; f Saver Heart Center, Unversity of Arizona, United States

**Keywords:** Heart Transplantation, ECMO, Survival, IABP

## Abstract

The United Network for Organ Sharing (UNOS) heart transplant allocation policy was changed in 2018. This study examines the impact of the change in UNOS heart transplant allocation policy on the use of temporary mechanical circulatory support (MCS) devices and post-transplant survival.

The analysis included a total of 26,481 patients listed and transplanted between January 2013 and June 2022. The results showed a decrease in waiting time for transplant after the policy change, indicating a successful reduction in waitlist time for high-priority status patients. However, the length of hospital stays from transplant to discharge increased following the policy change. The study also found an increase in the frequency of ECMO and IABP use both at the time of listing and at the time of transplant following the policy change.

Cumulative patient and graft survival at 1000 days decreased following the policy change (86.1 per cent versus 83.7 per cent at 1000 days, p = 0.002). However, the survival curves showed similar survival trends in the first 2 years, with late divergence in survival occurring after 2 years.

In conclusion the latest UNOS heart transplant allocation policy change led to a decrease in waiting times and an increase in the use of temporary MCS devices. There was a decrease in cummulative survival at 1000 days following the policy change.

## 1 Introduction

Cardiac transplantation remains the best long-term therapy for patients with medically refractory end-stage heart failure [1]. It is a durable treatment with 90 % one – year survival [2] and a median survival of 10 – 16 years [1], [3]. Eligibility to receive donor organs has been reviewed multiple times in national guidelines, with cardiogenic shock requiring either continuous intravenous inotropic support or mechanical circulatory support (MCS) regarded as top priority [4]. The prioritization of transplant candidates in terms of MCS may influence the use of mechanical devices in these patients.

The United Network for Organ Sharing (UNOS) heart transplant allocation policy was established in 1988 as 2 tier system and has undergone multiple revisions [5]. Prioritizing patients requiring temporary MCS, the current 6 – tier system aims to reduce waitlist mortality by expanding the high-priority status 1A to categories 1, 2, and 3. Following this, earlier reviews had reported varying survival outcomes and documented reduced waiting time, however, these reports were early and provided rates at 6 months [4], [6], [7], [8], [9]. These changes were attributed to an increase in the proportion of acutely ill patients transplanted, as evidenced by the increased use of extracorporeal membrane oxygenation (ECMO) and intra-aortic balloon pump (IABP). These studies were found to important limitation of informative early censoring and different results were seen in studies with longer follow up [10]. Possible explanations for the disparate results have been suggested to include: use of different analytical cohort, violation of noninformative censoring and differences in model building and covariates selection [11]. To date, little has been reported on the effect of the policy change on the use of these temporary mechanical support devices in association with cardiac transplant outcomes beyond 1 year.

The aim of this study was to evaluate trends in the frequency of use of temporary MCS – ECMO and IABP, before and after the most recent modification to the UNOS heart allocation policy; as well as the post-transplant survival at 1000 days while minimizing the above listed reasons for disparate results.

## 2 Materials and methods

This is a secondary data analysis of the record of all transplants done in all transplant centers across the United States of America. The publicly accessible deidentified dataset of United Network Organ Sharing (UNOS) was queried for heart transplant recipients listed for isolated orthotopic cardiac transplantation between January 1, 2013, and June 30, 2022. The UNOS STAR dataset includes de-identified patient-level data collected for every organ donor, transplant candidate, and transplant recipient in the United States. In addition, it contains information on recipient and donor centers, use of supplemental medications and MCS, as well as patient outcomes. On October 18, 2018, the new UNOS allocation policy for adult heart transplants went into effect.

Institutional review board waiver was obtained for this study and study was done in compliance with International Society for Heart and Lung Transplantation (ISHLT) ethics. Patterns of waitlist time, cumulative patient survival time at 1000 days, cumulative graft survival time at 1000 days, and frequency of temporary MCS (ECMO and IABP) in heart transplant recipients listed and transplanted before and after the most recent UNOS policy change were evaluated. The wait list data was censored between January 1, 2013, to October 17, 2018 (Period A); and October 18, 2018 to June 30, 2022 (Period B). The series means were used to replace > 5 % missing data. Data for survival analyses was censored at October 4, 2019 to allow for a minimum follow up period of 1000 days. Recipients of multiple organs and heart transplant recipients aged less than 18 years were excluded from the study.

Normality test was conducted using Q-Q Plots and confirmed using the Kolmogorov-Smirnov test. The median test and the Mann-Whitney *U* test were utilized to examine and confirm differences in waitlist time and length of stay from transplant to discharge. The autoregressive integrated moving average (ARIMA) modeling technique was used to forecast trends in IABP and ECMO utilization up to five years after the policy change. The log-rank test was used to compare survival distribution differences between groups.

Binary logistic regression analyses were performed to explore the relationship between the transplant period and use of temporary MCS among transplant recipients while controlling for the effect of age and sex. Categorical variables were compared using the χ^2^ test. Cumulative survival proportions at 1000 days were compared using Kaplan-Meier method. Results are reported as odds ratio (OR) with 95 % confidence intervals (CI) for categorical outcomes. Numerical outcomes are reported as median (IQR), or estimated means (EM) with 95 per cent confidence intervals (CI). A p-value less than 0.05 was considered to indicate statistical significance. Data gathering, cleaning, and analysis was performed in SPSS Statistical Software (IBM, Armonk, NY).

## 3 Results

Of the 26,481 patients waitlisted and transplanted during the study period, 16,326 were listed during Period A while 10,155 were listed during Period B (Table 1). The patients in period A were predominantly male (74 %) with a median (IQR) age of 56 (47 – 63) years. Also, the patients in Period B were predominantly male (72.3 %) with a median (IQR) age of 57 (46 – 63) years. The test for normality revealed a non-Gaussian distribution of waiting, patient survival, and graft survival times(p < 0.001). The median (IQR) waiting time, length of stay from transplant to discharge, the patient survival time and graft survival time for the entire study population were 70(17–––251) days, 16(11––25) days, 1077(363–––1834) days, and 1075(362–––1833) days respectively.

**Table 1:** Characteristics of transplant recipients before and after change in allocation policy.

**Characteristic**	**Period A (n = 17325)**	**Period B (n = 11764)**	**p-value**
**Age, median (IQR), y**	54.0 (36 – 62)	54.0 (37 – 62)	0.335
**Male gender**	12,284 (70.9 %)	8246 (70.1 %)	0.81

**Blood type**			
**A**	6698 (38.7 %)	4618(39.3 %)	0.002
**AB**	938 (5.4)	640 (5.4 %)	
**B**	2614 (15.1 %)	1837 (15.6 %)	
**O**	7041 (40.6 %)	4614 (39.2 %)	
**Others**	34 (0.2 %)	55 (0.5 %)	

**Race/ethnicity**			
**White**	10,733 (62 %)	6876 (58.4 %)	<0.001
**Black**	3858 (22.3 %)	2858 (24.3)	
**Hispanic**	1837 (10.5 %)	1404 (11.9 %)	
**Asian**	634 (3.7 %)	460 (3.9 %)	
**Other**	263 (1.5 %)	166 (1.4 %)	
**Recipient BMI (mean, SD)**	26.23 (±5.9)	26.37 (±5.8)	0.29

**Fig. 1:**
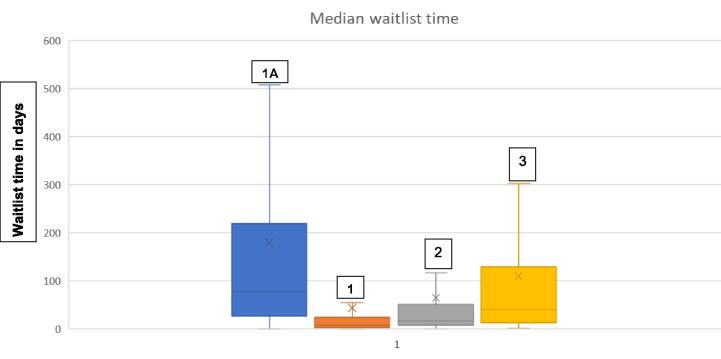
Q – Q Plot of Median (IQR) waiting time for patients in Status 1A, 1, 2 and 3.

There was a decrease in the waiting time to transplant following the change in allocation policy evidenced by the reduction in median (IQR) time on the cardiac transplant waitlist during Periods A and B [129 (36–371) vs 24 (8–86)] days, p < 0.001). Sub – group analysis of the waiting time for high – priority status showed a decreased median (IQR) waiting time from 93 (28 – 272) days for Status 1A in Period A to 9 (3 – 34), 21 (8 – 80) and 63 (17 – 273) days respectively for Status 1, 2 and, 3 in Period B. (Fig. 1) The length of the hospital stays from time of transplant to discharge increased following the change in allocation policy [median hospital stay of 16 (11 – 24) vs 17 (12–26) days, p < 0.001] (Table 2).

**Table 2:** Comparison of waiting time for the period 2013 – 2018 versus 2019–––2022.

**Variable**	**Period of observation**	**Median, IQR**	**p-value**
Waiting time (days)	Period A	99, 31 – 277	<0.001*
	Period B	28, 9 – 96	
Length of stay (days)	Period A Period B	16, 11 – 26 18, 12 – 27	<0.001

### 3.1 Patterns of temporary mechanical circulatory support use at transplant

There was an increase in the frequency of ECMO use at time of listing following the change in allocation policy (1.0 % vs 4.1 % p < 0.001). There was also an increase in the frequency of use of IABP at the time of listing between the two periods (4.6 % vs 17.4 % p < 0.001). This increase in temporary mechanical circulatory support use was also evident at the time of transplant with an increase in ECMO use from 0.9 % to 6.3 % (p < 0.001) and IABP from 7.5 % to 30.2 % (p < 0.001) (Table 3).

**Table 3:** Relationship between Transplant period and IABP and ECMO use among heart transplant patients.

**TIME PERIOD**	**IAPB USE AT TRANSPLANT**		**X^2^**	**p − value**
	**YES (%)**	**NO (%)**		
Period A	1,179 (6.8)	16,146 (93.2)	2094.78	< 0.001*
Period B	3,073 (26.1)	8,691 (73.9)		

**TIME PERIOD**	**ECMO USE AT TRANSPLANT**		**X^2^**	**p − value**
	**YES (%)**	**NO (%)**		
Period A	238 (1.4)	17,087 (98.6)	443.92	< 0.001*
Period B	679 (5.8)	11,085 (94.2)		

Regression analyses demonstrated that the odds of using IABP in patients transplanted during Period B was more than five times greater than the odds of using the IABP during period A (OR 5.36, 95 % Confidence Interval [CI] 4.99 – 5.76, p < 0.001, Supplementary file 1). Similarly, the odds of using ECMO at transplant was approximately seven times higher in period B than period A (OR 7.39, 95 % CI 6.17 – 8.87, p < 0.001, Supplementary file 2).

**Fig. 2:**
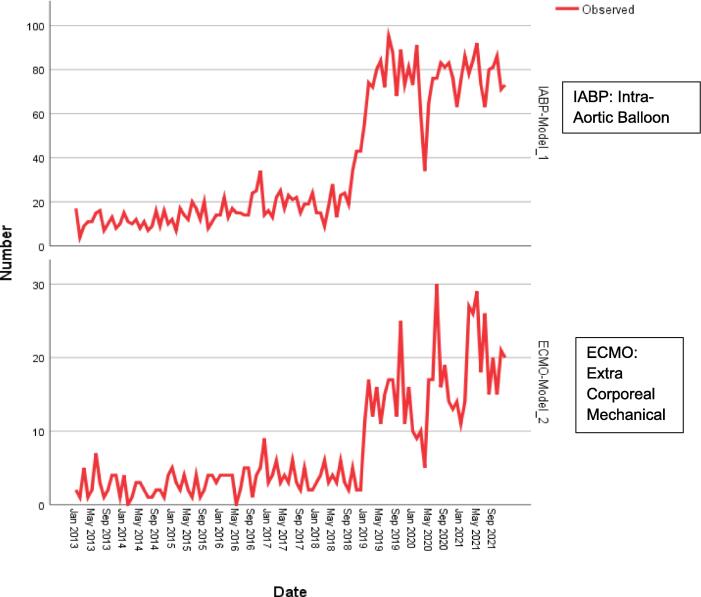
Frequency of use of IABP and ECMO at transplant from 2013 to 2021 among end-stage heart failure patients.

**Fig. 3:**
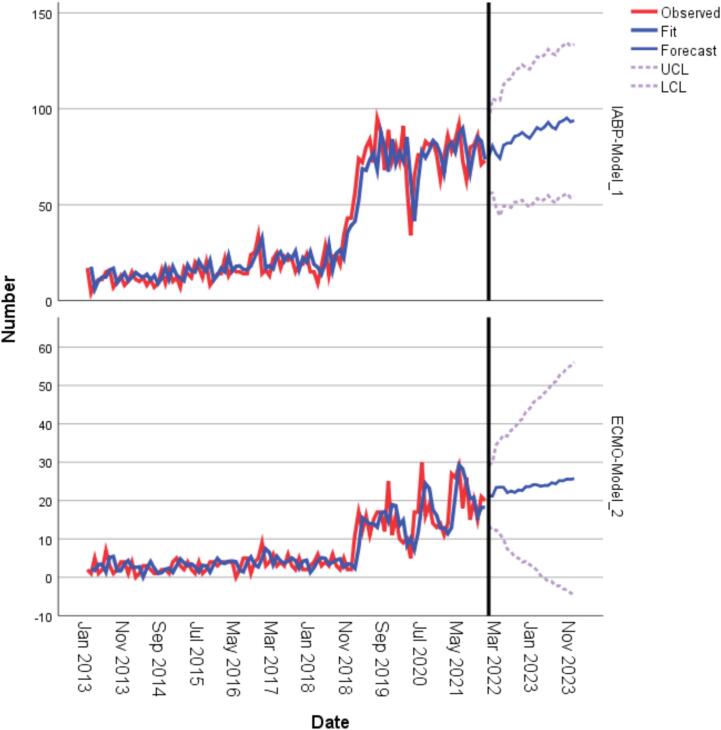
A forecast of the use of IABP and ECMO at transplant for the years 2022 and 2023 among end-stage heart failure patients.

The frequency of use of IABP and ECMO showed slight increase with time until after the policy change in 2018, following which there was an abrupt increase in the use of same procedures relative to the Period A period (Fig. 2). Additionally, quarterly variations were observed across the periods, with the number of procedures used peaking quarterly with intervening dips between successive quarters. The use of IABP and ECMO is expected to stay elevated up to 2023. (Fig. 3).

### 3.2 Relationship between transplant period and patient survival time

Out of the total study population of 26,481 patients, 17,605 patients were included in the survival analyses; this consisted of 15, 664 patients transplanted in Period A and 1941 patients transplanted in Period B. The median (IQR) age for Period A was 56 [47–63] years; the majority (73.7 %) were males. The median (IQR) age for Period B was 56 [45–63] years; also, the majority (69.8 %) were males.

**Fig. 4:**
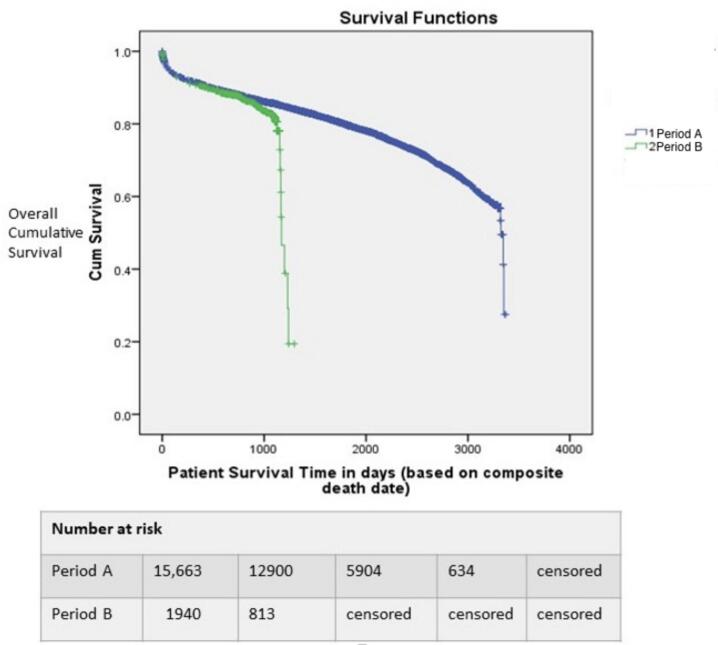
Comparison of cumulative patient survival proportion against time.

The median patient survival time for the entire study period was 1484 (1068 – 2194) days. The median survival time for Periods A and B were 1763 (1104 to 2254) and 777 (718 to 1089) respectively. Comparison of the cumulative patient survival against time following heart transplant revealed a higher survival trend among patients who received heart transplants prior to the new allocation policy (86.1 per cent versus 83.7 per cent at 1000 days, p = 0.002). (Fig. 4). Sub-group analysis of the trend in cumulative survival for high priority status 1A (Period A) and the equivalent three sub – groups (Status 1, 2 and, 3 combined) in Period B revealed a decrease in cumulative survival at 1000 days from 85.7 % to 83.9 % (p-0.003). Individually the cumulative survival rate at 1000 days for the new adult statuses 1, 2 and, 3 were not statistically different at 81.6, 84.6 and 83.4 % respectively (p = 0.21).

### 3.3 Relationship between transplant period and graft survival

The median graft survival time for the entire study period was 1482 (1065–––2193) days. Comparison of the cumulative graft survival against time following heart transplant revealed that graft survival was higher in patients who received heart transplants prior to the policy change than in those who received heart transplants after the allocation change (86.0 % vs 83.5 % at 1000 days, p = 0.003).

### 3.4 Relationship between ECMO use and patient’s survival time from 2013 to 2022

**Fig. 5:**
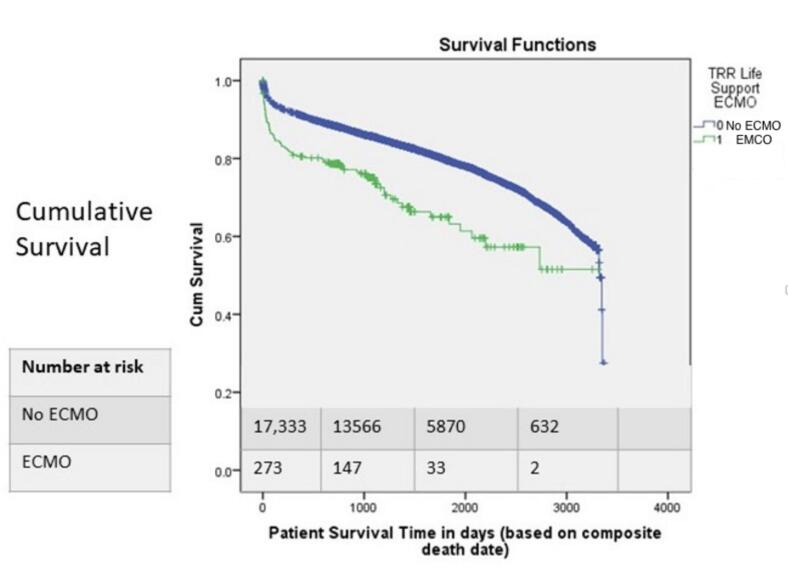
Comparison of cumulative survival proportion against time in the period 2013 – 2022 among end-stage heart failure patients who were placed on ECMO and those who were not.

Across the entire study period, the cumulative survival proportion against time was higher in the patients who were not placed on ECMO compared to those who were placed on ECMO. The cumulative survival at 1000 days was 86.0 % in patients who were not on ECMO versus 76.1 % in patients on ECMO, this observed difference was statistically significant Χ^2^ (1) = 32.84, p < 0.001. (Fig. 5).

### 3.5 Relationship between ECMO use and patient’s survival time from 2013 to 2018

The cumulative survival proportion against time was higher in the patients who were not placed on ECMO compared to those who were placed on ECMO during period A. The cumulative survival at 1000 days during period A was 86.3 % in patients not on ECMO versus 71.1 % in patients who were on ECMO at transplant, this observed difference was statistically significant Χ^2^ (1) = 30.09, p < 0.001.

### 3.6 Relationship between ECMO use and patient’s survival time in Period B

The cumulative survival at 1000 days was 83.9 % in patients who were not on ECMO at the time of transplant versus 81.0 % in patients who were on ECMO at the time of transplant, this observed difference was not statistically significant Χ^2^ (1) = 2.15, p = 0.14.

### 3.7 Relationship between IABP use and patient’s survival time from 2013 to 2022

The cumulative survival proportion against time was marginally higher in the patients who were not placed on IABP compared to those who were placed on IABP. The cumulative survival at 1000 days was 85.8 % in patients who were not on IABP at time of transplant and 84.0 % in patients who were on IABP at transplant, this difference was not statistically significant Χ^2^ (1) = 2.47, p = 0.116.

## 4 Discussion

Early review of the October 2018 UNOS cardiac transplant allocation change had reported improved waitlist outcomes, including lower waitlist mortality rates and higher rates of transplant [10]. This analysis examines the use of temporary mechanical circulatory support in the five years preceding and following the most recent allocation change and identifies an overall increase in use. The temporary MCS technologies that were assessed in this study include ECMO and IABP which the UNOS policy prioritized to class 1 and 2. We did not include the use of LVAD in the analysis. This exclusion was made to avoid confounding that may arise from the introduction of HeartMate 3 and discontinuation of HeartMate II which occurred during the study period. The implementation of the most recent change in UNOS cardiac transplant allocation policy led to an increase in the use of temporary mechanical circulatory support, which can be attributed to practice changes in response to the new policy.

Cardiac transplantation remains the standard of care for many patients with irreversible end-stage pump failure who are refractory to medical treatment [1]. This is due to the durability of the treatment with long term outcomes which favor transplantation over competing options [12]. The availability of donor organs is the most limiting factor in providing this treatment hence allocation of this scarce resource has been to those most in need [2]. The aim of the latest change in UNOS policy was to ensure an equitable allocation of donor organs to patients who are in most urgent need [13]. This study indicates that the 2018 UNOS allocation policy change has been successful in achieving its goal of reducing time to transplant for the high – priority status patients evidenced by the decreased waiting times for transplant candidates.

The increased use of MCS has been suggested to reflect prioritization of acutely ill patients for organ transplantation, [6] this is a departure from the pre-policy trend in which these patients accounted for the high waitlist mortality rate [14]. This may be considered a successful alignment of the UNOS policy with the Department of Health and Human Services’ Final Rule which mandates the “best use” of donor organs [12], [15]. However, there had been concerns that this shift in physician- and hospital- level practice would compromise outcome [16].

The spike in frequency of use of temporary MCS among transplant patients following the implementation of the new policy suggests that listing patterns for heart transplants have changed. The occurrence of changes in listing patterns following policy changes had previously been reported and were not explained by patient characteristics. In particular, the authors were concerned that programs are overtreating less urgent candidates in order to obtain high priority Status [17]. In a single center review [18] in which the most common listing status changed from status 1B to 2 following the implementation of the policy change, the increased use of IABP as a bridging strategy was attributed to increased insertion of IABP via the axillary approach. It is noteworthy that description of alternative IABP insertion routes [19] predates this UNOS policy change and the spike in frequency of MCS use observed in this study. Percutaneous approach to vascular access for MCS, although used in unstable as bridge to transplant, [20], [21], [22] may be occasioned with catastrophic complications, [23] and are unlikely to account for the spike in the frequency of use of temporary MCS observed in this study. It was not possible to determine the predominant approach for insertion of IABP in this study.

The survival curves in this study demonstrate similar survival trends for the first two years, with divergence occurring after 2 years. This contrasts with an earlier finding of diverging survival in the first six months [6]. While the trend towards lower survival reported in an earlier review (done at 1 year of the new policy) may be attributable to drastic shift in patient characteristics [10] in addition to increase in documented risk factors of post-transplant mortality [24], [25], these factors do not explain our finding of similar initial survival and differing late survival. Our finding of the survival curve diverging after the second year suggests that the survival trend in the new era initially approximates that in the old era until towards the end of the follow up period. This trend towards a reduction in divergence of the survival trend with time verifies a probable finding from a previous review done at one year which suggest that the reduction in survival was perhaps less substantial than was anticipated [10]. It is noteworthy that 4 out of 5 previous reviews reported decrease in unadjusted post-transplant survival at six months, [4], [6], [7], [8] the only review which reported no difference in unadjusted post-transplant survival at 6 months had a relatively later follow up period (November versus June/September 2019) [9]. These differences in reported outcomes may be due to ascertainment bias resulting from lag in the reporting systems of the UNOS database. Deaths are reported sooner than survival data; for instance, survivors who are alive at 11 months post-transplant may be censored at 6 months until 1 year data is reported however a mortality event at 7 months is reported immediately thereafter. This may explain why, in all reviews, the survival in the new era is lower proximal to the time of review.

A cardiac transplant scoring system which prioritizes post-transplant survival and quality of life [26] may be a recommended approach to optimize “best use”. In addition, the inclusion of objective evidence of hemodynamic compromise or adverse events in the algorithm will prevent the adoption of practice changes that do not reflect candidate characteristics [17]. Lessons may be drawn from the implementation of such scoring system for the allocation of lungs, [27], [28], [29] liver [30] and kidneys [31]. Early experience with cardiac allocation scoring as it is practiced elsewhere, indicates a predictable outcome after transplantation [32]. A combination of post-transplant survival and quality of life should determine allocation of organs.

A limitation of this study is that it assumes that reporting of survival data is real time; that a minimum follow-up period of 1000 days is adequate to assess cumulative survival at 1000 days. Further studies should be done to determine the lag in survival reporting. Also, this study does not include the factor of COVID-19 pandemic on the survival of these patients who are immunocompromised.

## 5 Conclusion

The most recent UNOS policy change has been successful in reducing waitlist times. Implementation of the new policy was followed by a spike in use of MCS. The survival trend curves showed similar survival up to 2 years post-transplant; thereafter, the survival was lower in the new era as determined by cumulative survival at 1000 days.

## Data availability statement

The de-identified data used for this article may be accessed by the public from the data website (https://optn.transplant.hrsa.gov/data/view-data-reports/request-data/).

## Ethical statement

This research underwent review and approval by University of Arizona Institutional Review Board (IRB). We ensured that the work described herein was conducted in strict adherence to the World Medical Association's Code of Ethics for experiments involving humans (Declaration of Helsinki). Informed consent from individual patients was waived by the IRB due to the retrospective nature of the analysis and use of de-identified data.

## Author contributions

CPE did the data analysis and prepared the manuscript. HM conceptualized the study and prepared the manuscript. MR reviewed the manuscript and approved the final manuscript. DA discussed the results, reviewed the manuscript and approved the final draft. TK reviewed the manuscript and approved the final draft. RH reviewed the manuscript and approved the final draft.

## CRediT authorship contribution statement

**Chidiebere Peter Echieh:** Writing – original draft, Visualization, Software, Project administration, Methodology, Formal analysis, Conceptualization. **Mohammad Hamidi:** Validation, Formal analysis. **Michael P. Rogers:** Writing – review & editing, Validation, Formal analysis. **Deepak Acharya:** Writing – review & editing. **Toshinobu Kazui:** Writing – review & editing. **Robert L. Hooker:** Writing – review & editing.

## Declaration of competing interest

The authors declare that they have no known competing financial interests or personal relationships that could have appeared to influence the work reported in this paper.

## References

[1] McCartney S.L., Patel C., Del Rio J.M. (2017). Long-term outcomes and management of the heart transplant recipient. Best Pract. Res. Clin. Anaesthesiol..

[2] Moayedi Y., Fan C.P.S., Cherikh W.S. (2019). Survival outcomes after heart transplantation: does recipient sex matter?. Circ Hear Fail.

[3] Khush K.K., Cherikh W.S., Chambers D.C. (2018). The international thoracic organ transplant registry of the international society for heart and lung transplantation: thirty-fifth adult heart transplantation report—2018; focus theme: multiorgan transplantation. J Hear Lung Transplant.

[4] Cogswell R., John R., Estep J.D. (2020). An early investigation of outcomes with the new 2018 donor heart allocation system in the United States. J Hear Lung Transplant.

[5] Shore S., Golbus J.R., Aaronson K.D. (2020). Changes in the United States adult heart allocation policy challenges and opportunities. Circ. Cardiovasc. Qual. Outcomes.

[6] Kilic A., Hickey G., Mathier M.A. (2020). Outcomes of the first 1300 adult heart transplants in the United States after the allocation policy change. Circulation.

[7] Jawitz O.K., Mhs M.F., Raman V. (2020). Reassessing recipient mortality under the new heart allocation system. JACC Hear Fail.

[8] Trivedi J.R., Slaughter M.S. (2020). “ Unintended ” consequences of changes in heart transplant allocation policy : impact on practice patterns. ASAIO J..

[9] Goff R.R., Uccellini K., Lindblad K. (2020). A change of heart: preliminary results of the US 2018 adult heart allocation revision. Am. J. Transplant..

[10] Kilic A., Mathier M.A., Hickey G.W. (2021). Evolving trends in adult heart transplant with the 2018 heart allocation policy change. JAMA Cardiol..

[11] Varshney A.S., Hirji S.A., Givertz M.M. (2020). Outcomes in the 2018 UNOS donor heart allocation system: a perspective on disparate analyses. J Hear Lung Transplant.

[12] Health Resources and Services Administration (HRSA) D of H and HS (HHS). Organ Procurement and Transplantation Network. Final Rule. Fed Regist., 2013. 11010703

[13] OPTN|UNOS Thoracic Organ Transplantation Committee, OPTN|UNOS Policy Department. Proposal to Modify the Adult Heart Allocation System., 2016.

[14] Blackstone E.H., Rajeswaran J., Cruz V.B. (2018). Continuously updated estimation of heart transplant waitlist mortality. J. Am. Coll. Cardiol..

[15] Penhoet E.D., Duan N., Dubler N.N. (1999). Organ procurement and transplantation: assessing current policies and the potential impact of the DHHS final rule committee on organ procurement and transplantation policy. Inst. Med...

[16] Varshney A.S., Berg D.D., Katz J.N. (2020). Use of temporary mechanical circulatory support for management of cardiogenic shock before and after the united network for organ sharing donor heart allocation system changes. JAMA Cardiol..

[17] Parker W.F., Garrity E.R., Fedson S., Churpek M.M. (2017). Trends in the use of inotropes to list adult heart transplant candidates at status 1A. Circ Hear Fail.

[18] Liu J, Yang BQ, Itoh A et al. Impact of New UNOS Allocation Criteria on Heart Transplant Practices and Outcomes. 2020:1–7. 10.1097/TXD.0000000000001088 PMC7738116 33335981

[19] Burack J.H., Uceda P., Cunningham J.N. (1996). Transthoracic intraaortic balloon pump: a simplified technique. Ann. Thorac. Surg..

[20] Estep J.D., Cordero-Reyes A.M., Bhimaraj A. (2013). Percutaneous placement of an intra-aortic balloon pump in the left axillary/subclavian position provides safe, ambulatory long-term support as bridge to heart transplantation. JACC Hear Fail.

[21] Onorati F., Bilotta M., Pezzo F. (2006). Transbrachial insertion of a 7.5-Fr intra-aortic balloon pump in a severely atherosclerotic patient. Crit. Care Med..

[22] Bundhoo S., O’Keefe P.A., Luckraz H. (2008). Work in progress report - Assisted circulation: extended duration of brachially inserted intra-aortic balloon pump for myocardial protection in two patients undergoing urgent coronary artery bypass grafting. Interact. Cardiovasc. Thorac. Surg..

[23] Alvarez C.K., Alvarez Villela M., Wiley J.M. (2020). Axillary intra-aortic balloon pump migration into the left ventricle during peripheral venoarterial extracorporeal membrane oxygenation support. Circ Hear Fail.

[24] Russo M.J., Iribarne A., Hong K.N. (2010). Factors associated with primary graft failure after heart transplantation. Transplantation.

[25] Fukuhara S., Takeda K., Kurlansky P.A. (2018). Extracorporeal membrane oxygenation as a direct bridge to heart transplantation in adults. J. Thorac. Cardiovasc. Surg..

[26] Faitot F., Michard B., Artzner T. (2020). Organ allocation in the age of the algorithm: avoiding futile transplantation - utility in allocation. Curr. Opin. Organ Transplant..

[27] Davis S.Q., Garrity E.R. (2007). Organ allocation in lung transplant. Chest.

[28] Gottlieb J. (2017). Lung Allocation. J. Thorac. Dis..

[29] Braun A.T., Dasenbrook E.C., Shah A.S. (2015). Impact of lung allocation score on survival in cystic fibrosis lung transplant recipients. J. Hear Lung Transplant.

[30] Graziadei I. (2006). Liver transplantation organ allocation between child and MELD. Wien. Med. Wochenschr..

[31] Johnson A.P., Price T.P., Lieby B. (2016). Dual kidney allocation score: a novel algorithm utilizing expanded donor criteria for the allocation of dual kidneys in adults. Ann. Transplant..

[32] Claes S., Berchtold-Herz M., Zhou Q. (2017). Towards a cardiac allocation score: a retrospective calculation for 73 patients from a German transplant center. J. Cardiothorac. Surg..

