# Supplementary material for: Survival trends in heart transplant patients supported on ECMO and IABP: A 10-year UNOS database analysis
Source: Int J Cardiol Heart Vasc. 2024 Aug 13;54:101486. doi: 10.1016/j.ijcha.2024.101486 (PMC11367637; doi:10.1016/j.ijcha.2024.101486)
Supplement: Supplementary Data 1 [file mmc1.docx]

**Supplementary file 1: Binary logistic regression analysis of the association between time period and IABP use**

| **Independent variables** | **Frequency** | **aOR** | **P - value** | **95% CI** |
| --- | --- | --- | --- | --- |
| **Age group** |  |  |  |  |
|  |  |  |  |  |
| 18 – 39 | 4,215 | 1 |  |  |
| 40 – 59 | 11,862 | 1.16 | 0.005* | 1.05 – 1.29 |
| 60 – 79 | 10,404 | 1.31 | < 0.001* | 1.18 – 1.46 |
| **Sex** |  |  |  |  |
| Female | 7,064 | 1 |  |  |
| Male | 19,417 | 1.12 | 0.004* | 1.03 – 1.21 |
| **Time period** |  |  |  |  |
| Period A | 16,326 | 1 |  |  |
| Period B | 10,155 | 5.36 | < 0.001* | 4.99 – 5.76 |

**Supplementary file 2: Binary logistic regression analysis of the association between time period and ECMO use**

| **Independent variables** | **Frequency** | **aOR** | **P - value** | **95% CI** |
| --- | --- | --- | --- | --- |
| **Age group** |  |  |  |  |
|  |  |  |  |  |
| 18 – 39 | 4,215 | 1 |  |  |
| 40 – 59 | 11,862 | 3.12 | < 0.001* | 2.57 – 3.80 |
| 60 – 79 | 10,404 | 1.58 | < 0.001* | 1.32 – 1.89 |
| **Sex** |  |  |  |  |
| Female | 7,064 | 1 |  |  |
| Male | 19,417 | 1.27 | 0.004* | 1.08 – 1.51 |
| **Time period** |  |  |  |  |
| Period A | 16,326 | 1 |  |  |
| Period B | 10,155 | 7.34 | < 0.001* | 6.17 – 8.87 |
